# Fibroblast depletion reveals mammalian epithelial resilience across neonatal and adult stages

**DOI:** 10.1101/2025.07.29.667423

**Published:** 2025-07-31

**Authors:** Isabella M. Gaeta, Shuangshuang Du, Clémentine Villeneuve, David G. Gonzalez, Catherine Matte-Martone, Smirthy Ganesan, Deandra Simpson, Jessica L Moore, Chen Yuan Kam, Sara Gallini, Haoyang Wei, Fabien Bertillot, Dagmar Zeuschner, Lauren E. Gonzalez, Kaelyn D Sumigray, Sara A Wickström, Valentina Greco

**Affiliations:** 1 Department of Genetics, Yale School of Medicine, New Haven, CT 06510 USA.; 2 Department of Cell and Tissue Dynamics, Max Planck Institute for Molecular Biomedicine, 48149 Münster, Germany.; 3 Stem Cells and Metabolism Research Program, Faculty of Medicine, University of Helsinki, 00290 Helsinki, Finland.; 4 Departments of Cell Biology and Dermatology, Yale Stem Cell Center, Yale Cancer Center, Yale School of Medicine, New Haven, CT 06510 USA.; 5 Howard Hughes Medical Institute (HHMI), Chevy Chase, MD, USA

## Abstract

Regenerative organs, like the skin, depend on niche-stem cell interactions that sustain continuous cellular turnover. In cell culture, skin fibroblasts promote epidermal stem cell proliferation and differentiation. Yet, it remains elusive how fibroblasts regulate epidermal stem cell behaviors and differentiation *in vivo*. Here, we asked how fibroblast depletion may impact epidermal stem cell proliferation in the context of adult homeostasis. Surprisingly, we find that significant depletion of fibroblast density does not affect epidermal stem cell proliferative capacity during adult stages *in vivo*. We next probed earlier neonatal stages when skin is actively remodeling but found no change in epidermal stem cell proliferative capacity following fibroblast depletion. These results demonstrate that across different ages, epidermal stem cell proliferative capacity can persist in the face of a largely reduced fibroblast population. Interestingly, neonatal fibroblast depletion does not significantly reduce their secreted collagen I density but affects basement membrane mechanics and epidermal stem cell delamination. Despite these changes to basement membrane mechanics and delamination, the skin continues to maintain its protective barrier function. Thus, our work demonstrates the skin regenerative program employs robust compensatory mechanisms in the face of fibroblast depletion to maintain functional capacity.

## Introduction

Regenerative organs undergo continuous cell turnover, which involves the production of new cells to replace those that are lost in a niche-regulated manner^1–3^. Skin is an ideal model system to study epithelial regenerative capacity in relationship to its mesenchymal niche because of its continual turnover over the lifetime of an organism and its unique accessibility. In the mammalian skin epidermis, new cells are generated within the basal epidermal stem cell layer by proliferation and move upward by delamination to overlying epithelial layers that maintain the skin barrier. Proper regulation of proliferation and delamination is crucial for maintaining cell density and skin function during homeostasis. The epidermis is in contact with an underlying dermal layer, which is rich in mesenchymal fibroblasts. Co-culture studies demonstrate fibroblasts act as a niche to promote epidermal stem cell proliferation and differentiation^4–7^. Additionally, in skin appendages such as the hair follicle, specialized dermal papilla fibroblasts secrete factors that activate hair follicle epithelial stem cell proliferation^8,9^ and are required for hair follicle growth^10^ and specification of hair epithelial cell type and size^11^. However, how fibroblasts support proliferative demands and differentiation of epithelial cells residing in the epidermal stem cell layer is not understood *in vivo*.

Here, we investigate the relationship between fibroblasts and dynamic turnover in the skin epidermis using mouse models. We asked how fibroblast depletion affects stem cell proliferative capacity during homeostasis in adult mice and demonstrate that fibroblast density depletion *in vivo* does not lead to changes in proliferation in the epidermal stem cell layer. Similarly, fibroblast depletion at neonatal stages – a stage of organ expansion and maturation - also does not affect stem cell proliferative capacity. We find that the major structural component secreted by fibroblasts, collagen I, is not significantly reduced following fibroblast depletion during neonatal stages, however basement membrane (BM) mechanics are perturbed. Moreover, while we find a mild reduction in epidermal stem cell delamination, the barrier-protective function of the skin epidermis remains intact following fibroblast depletion during neonatal stages. Together, this study reveals that epidermal stem cell proliferation persists in response to fibroblast depletion, suggesting that the skin employs mechanisms to compensate for fibroblast loss during both skin maturation and homeostasis.

## Results

### Epidermal stem cell proliferation persists despite fibroblast depletion during adult homeostasis

**Figure 1.**
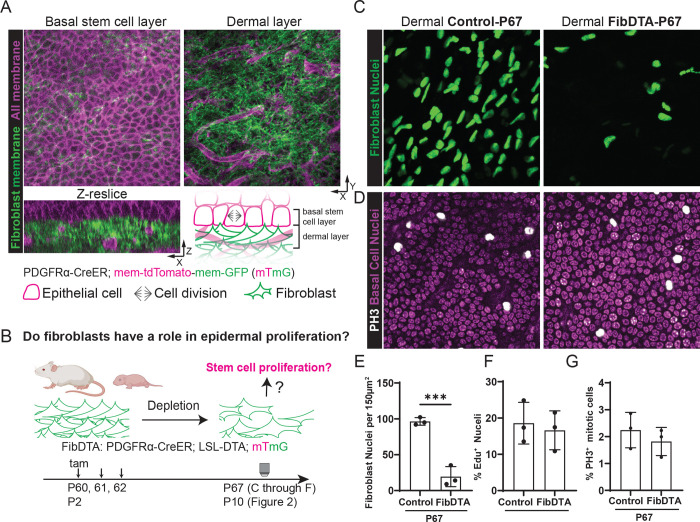
Epidermal stem cell proliferation persists despite fibroblast depletion during adult homeostasis A) Live imaging of the non-hairy paw skin of PDGFRα-CreER; mTmG mice at P10 with fibroblast membrane GFP in green and all cell membrane labeled by tdTomato in magenta. Top left panel: max projection of the epidermal stem cell layer 6 μm above the dermal second harmonic generation (SHG) signal as defined in Imaris. Top right panel: max projection of signal 10 μm into the dermal layer as defined in Imaris. Box widths = 150 μm. Bottom left panel: resliced image of top panels with 1 μm z steps. Resliced image = 150 μm × 50μm. Bottom right panel: cartoon representation of the proximity between the basal epidermal stem cell layer and dermal fibroblasts with their designated icons shown at the bottom. (B) Schematic of the FibDTA model in adult and neonatal mice (PDGFRα-CreER; LSL-DTA; mTmG). Tamoxifen was given at P60 for adults and P2 for neonates (Figure 2) to induce fibroblast density depletion. Tissue collection or two-photon imaging or of the paw skin was done at P67 or P10 for neonates and adults respectively. (C) Representative whole mount images of fibroblast nuclei (PDGFRα-H2BGFP) signal in green. Max projection of 10 μm into the dermis of control (PDGFRα-H2BGFP; LSL-DTA) and FibDTA (PDGFRα-CreER; LSL-DTA; PDGFRα-H2BGFP) mice at P67. Box widths = 150 μm. (D) Representative whole mount images of epidermal stem cells stained with PH3 in white and Hoechst in magenta from P67 control (PDGFRα-H2BGFP; LSL-DTA) and FibDTA (PDGFRα-CreER; LSL-DTA; PDGFRα-H2BGFP) mice. Box widths = 150 μm. (E) Quantification of fibroblast number identified by the PDGFRα-H2BGFP signal in control and FibDTA mice in adult. Graph represents the average number of fibroblast nuclei in the upper dermal layer (10 μm into the dermis) within a 150 μm^2^ area with SD from n = 3 control (LSL-DTA; PDGFRα-H2BGFP) and 3 FibDTA (PDGFRα-CreER; LSL-DTA; PDGFRα-H2BGFP) mice. ^***^p = 0.0009, unpaired, two-sided, Student’s t-test. (F) Quantification of %Edu+ epidermal stem cells in control and FibDTA mice at P67 within a 200 μm^2^ area. n = 3 control (mTmG;LSL-DTA) and 3 FibDTA (PDGFRα-CreER;mTmG;LSL-DTA). p = 0.6874 ,ns, unpaired, two-sided, Student’s t-test G) Quantification of proliferation events in the epidermal stem cell layer in control and FibDTA adult mice. Graph represents the average number of PH3-positive cells within a 150 μm^2^ area with SD from n = 3 control (LSL-DTA; PDGFRα-H2BGFP) and 3 FibDTA (PDGFRα-CreER; LSL-DTA; PDGFRα-H2BGFP) mice. p = 0.4313, ns, unpaired, two-sided, Student’s t-test.

In cell culture, fibroblasts promote epidermal stem cell proliferation and expansion^4–7^. To investigate the potential impact of fibroblasts on the behavior and function of epidermal stem cells in live mice, we employed a skin model – the paw – that lacks remodeling associated with skin appendages and allows maximal surface area of the epidermis to be examined^12,13^. To differentially visualize fibroblasts and epidermal cells, we utilized an inducible Cre ER line that is largely expressed in the fibroblast population (PDGFRα-CreER) in combination with a fluorescent reporter (Rosa-membrane-tdTomato-membrane-GFP, abbreviated mTmG^14^). Fibroblasts, labeled by membrane GFP, are present throughout the dermal space and interface with epidermal cells labeled by membrane tdTomato and organized in a hexagonal appearance (Figure 1A). To investigate the extent to which fibroblasts impact epidermal stem cell proliferation *in vivo*, we reduced fibroblast density in adult mice using a mouse model expressing diphtheria toxin fragment A (DTA) under the control of PDGFRα-CreER (LSL-DTA; PDGFRα-CreER, referred to as FibDTA) (Figure 1B). To track fibroblasts, we used both a fluorescent membrane reporter (either mTmG or LSL-tdTomato) as well as a nuclear reporter independent of Cre regulation (PDGFRα-H2BGFP). We showed first that PDGFRα-CreER and PDGFRα-H2BGFP label equivalent populations (Supplemental Figure 1). Second, through administration of 100 ug/g tamoxifen for 3 days, postnatal days 60–63 (P60–63), we detected a ~70% depletion of fibroblast density in adult FibDTA at P67 compared to control littermates by imaging of fibroblast nuclei (quantification of the top 10μm of the dermis, Figure 1C and E). Additional marker analyses showed that fibroblasts were depleted across all fibroblast subtypes (CD26^+^ Sca1^−^, CD26^−^Sca1^+^ or CD26^+^Sca1^+^; Supplementary Figure 2A and B).

To investigate the impact of fibroblast depletion on epidermal proliferation, we performed a pulse of 5-ethynyl-2’-deoxyuridine (Edu) followed by a 6-hour chase to detect cells that have gone through S phase. Remarkably, we found that in the epidermal stem cell layer the number of cells undergoing DNA synthesis per total epidermal stem cells was similar when comparing fibroblast depleted conditions to controls (Figure 1F and Supplementary Figure 3E). Moreover, as Edu incorporation marks broader phases of the cell cycle, we more specifically measured cells undergoing proliferation in M phase by phospho-histone H3 staining (PH3). We found that the percentage of total epidermal stem cells undergoing mitotic events does not change in fibroblast depleted mice, nor do the density of the epidermal stem cells when compared to control mice (Figure 1D and G, Supplementary Figure 2E). These findings demonstrate that adult epidermal stem cells retain the capacity to proliferate despite significant fibroblast depletion during adult homeostasis.

### Epidermal stem cell proliferation persists despite fibroblast depletion during neonatal development

**Figure 2.**
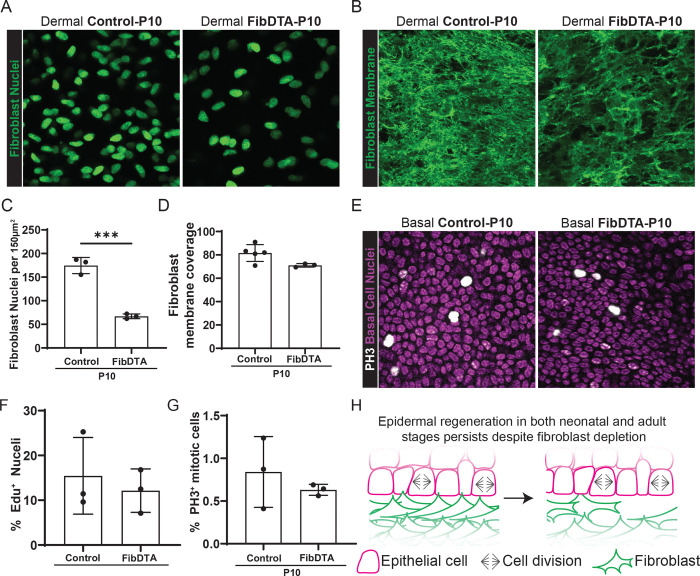
Epidermal stem cell proliferation persists despite fibroblast depletion during neonatal development (A) Representative neonatal fibroblast nuclei (PDGFRα-H2BGFP) signal in green from live mouse imaging. Max projection of 10 μm into the dermal layer of control (LSL-DTA; PDGFRα-H2BGFP) and FibDTA (PDGFRα-CreER; LSL-DTA; PDGFRα-H2BGFP) mice at P10. Box widths = 150 μm (B) Max projection of fibroblast membrane signal from the upper dermal layer of live mice defined in Imaris (10 μm into the dermis) from control (PDGFRα-CreER; mTmG) and FibDTA (PDGFRα-CreER; mTmG; LSL-DTA) mice at P10. Box width = 150 μm. (C) Quantification of fibroblast number identified by the PDGFRα-H2BGFP signal in control and FibDTA in P10 mice. Graph represents the average number of fibroblast nuclei in the upper dermal layer (10 μm into the dermis) within a 150 μm^2^ area with SD from n = 3 control (LSL-DTA; PDGFRα-H2BGFP) and 3 FibDTA (PDGFRα-CreER; LSL-DTA; PDGFRα-H2BGFP) mice. ^***^p = 0.0005, unpaired, two-sided, Student’s t-test. (D) Quantification of the fibroblast membrane coverage in FibDTA mice compared to control mice at P10. Graph represents the percent coverage of membrane GFP signal max projection in the upper dermal layer (10 μm into the dermis) within a 150 μm^2^ area with SD from n = 5 control mice and 3 FibDTA mice p = 0.0508, ns, unpaired, two-sided, Student’s t-test. (E) Representative whole mount images of epidermal stem cells stained with PH3 in white and Hoechst in magenta from P10 control (PDGFRα-CreER) and FibDTA (PDGFRα-CreER; LSL-DTA) mice. The isolation of the epidermal stem cell layer relative to the other layers was determined by the small and compact nuclei of basal epidermal stem cells stained with Hoechst and their proximity to SHG signal. Box widths = 150 μm. (F) Quantification of %Edu+ epidermal stem cells in control and FibDTA mice at P10 within a 200 μm^2^ area as shown in H. n = 3 control (PDGFRα-H2B-GFP; LSL-DTA; or LSL-DTA) and 3 FibDTA (PDGFRα-CreER; LSL-DTA; PDGFRα-H2B-GFP or PDGFRα-CreER; LSL-DTA) mice. p= 0.5837, ns, unpaired, two-sided, Student’s t-test. (G) Quantification of proliferation events in the epidermal stem cell layer in control and FibDTA P10 mice. Graph represents the average number of PH3-positive cells within a 216 μm^2^ area with SD from n = 3 control (PDGFRα-CreER) and 3 FibDTA (PDGFRα-CreER; LSL-DTA) mice. p = 0.4357, ns, unpaired, two-sided, Student’s t-test. (H) Schematic summary of the impact of fibroblast depletion on epidermal stem cell regeneration in neonatal and adult mice.

We reasoned that during adulthood epidermal stem cells and their niche may have established multiple components to support epithelial proliferation. This prompted us to ask whether proliferative capacity of epidermal stem cells is more reliant on fibroblasts during neonatal development, when the organ is both expanding and maturing to reach adult size. To mitigate systemic effects of fibroblast depletion we began by locally inducing fibroblast depletion in the skin at P2 through topical administration of 4-hydroxy tamoxifen (4-OHT). Administration of 20mg/mL 4-OHT directly to paw skin for two consecutive days (P2 and P3) resulted in a ~30% depletion of fibroblast density (Supplementary Figure 3A and C) and no change in epidermal stem cell density at P10 (Supplementary Figure 3B and D). To increase the extent of neonatal depletion similar to experiments in adult mice, we turned to systemic delivery of 80 μg/g tamoxifen at P2, achieving a ~60% depletion of fibroblast density at P10 across subtypes measured by live imaging and FACS (Figure 2A and C, Supplementary Figure 2C, D and F). Interestingly, despite a dramatic reduction of fibroblasts, quantification of the fibroblast membrane coverage (LSL-DTA; PDGFRα-CreER; mTmG) showed no significant change (Figure 2B and D). We note that systemic depletion of fibroblasts leads to significantly reduced weight of fibroblast depleted neonatal mice (Supplementary Figure 2G).

To determine whether systemic fibroblast depletion impacts epidermal stem cell proliferation at neonatal stages, we again measured DNA synthesis and mitotic events by using a 6-hour Edu chase and PH3 staining respectively. We found that the number of cells undergoing DNA synthesis per total epidermal stem cells was similar when comparing fibroblast depleted conditions to controls (Figure 2F and Supplementary Figure 3F). Additionally, we find that the percentage of total epidermal stem cells undergoing mitotic events also does not change between control and fibroblast depleted neonatal mice (Figure 2E and G).

Large population depletion can cause immune reactions^15^, which may also be contributing as a compensatory mechanism to the sustainment of epidermal proliferation. We showed that our DTA model did not cause immune cell recruitment locally to paw tissues (Supplementary Figure 4 A–D) nor an increase in circulating cytokine levels in serum (Supplementary Figure 4E). Moreover, we did not detect gross architectural changes in nearby tissues, such as the vasculature capillary plexus upon fibroblast depletion (Supplementary Figure 4F and G).

Taken together, these findings demonstrate that epidermal stem cells retain their capacity for proliferation upon fibroblast depletion in neonatal contexts, paralleling the results obtained in the adult context (Figure 2H). These results suggest that multiple sources support epidermal stem cell growth upon fibroblast loss *in vivo*.

### Neonatal fibroblast depletion alters basement membrane mechanics and epidermal delamination with no impact on collagen density or tissue function

**Figure 3.**
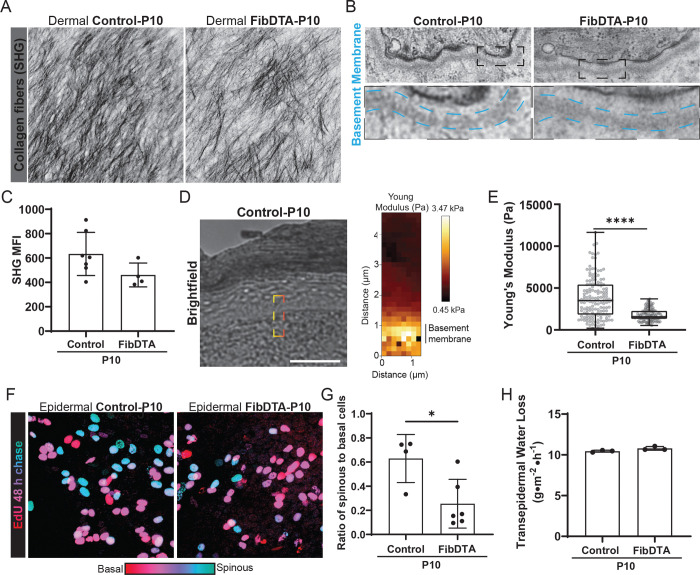
Neonatal fibroblast depletion alters basement membrane mechanics and epidermal delamination with no impact on collagen density or tissue function (A) Representative max projection of the SHG signal within 10 μm into the dermis of live mice as defined in Imaris, revealing the structure of collagen fibers from P10 control (mTmG; PDGFRα-CreER or mTmG; LSL-DTA or LSL-DTA), FibDTA (mTmG; PDGFRα-CreER; LSL-DTA). (B) Representative transmission electron micrographs of basement membrane in control (PDGFRα-CreER), FibDTA (PDGFRα-CreER; LSL-DTA) paw skin collected at P10. Dashed boxes indicate zoom regions to highlight basement membrane, outlined in cyan. Top box widths = 1.5μm. Zoom box widths = 0.5μm (C) Quantification of the collagen fiber density in the upper dermal layer in control and FibDTA, mice at P10. Graph represents average MFI of SHG signal within a 150 μm^2^ area with SD from n = 7 control mice (mTmG; PDGFRα-CreER or mTmG; LSL-DTA or LSL-DTA), and n = 4 FibDTA mice (mTmG; PDGFRα-CreER; LSL-DTA). p = 0.1101, ns, unpaired, two-sided, Student’s t-test. (D) Representative brightfield image produced during AFM measurements and corresponding heat map of young modulus to measure basement membrane stiffness. Red dashed box indicates representative region of young modulus heat map measurement. Scale bar = 50 μm (E) Tukey box and whiskers plot of Young’s elastic moduli from atomic force indentation spectroscopy measurements in skin BM regions from P10 control and FibDTA mice. Outliers were identified using the ROUT method and removed, resulting in n=147 force curves from 3 control (LSL-DTA or Col1a1 fl/fl) mice and n = 122 force curves from 3 FibDTA (PDGFRα-CreER; LSL-DTA) mice. ****p = <0.0001, two tailed, Kolmogorov-Smirnov test. (F) Images showing EdU whole mount staining after 48-hour pulse-chase from P8 to P10 color-coded by depth with red (epidermal basal cells) closer to the dermis and blue (epidermal spinous cells) further into the epidermis from P10 control (LSL-DTA) and FibDTA (PDGFRα-CreER; LSL-DTA) mice. Relative locations of EdU+ cells to the epidermal stem cell layer are noted, and cells are categorized as EdU+ spinous cells versus EdU+ basal cells. Box width = 140 μm. G) Quantification of the rate of delamination in control and FibDTA mice at P10. Graph represents the average ratio of EdU+ spinous to basal cells with SD from n = 4 control (LSL-DTA) and 6 FibDTA (PDGFRα-CreER; LSL-DTA) mice at P10. *p = 0.0203, unpaired, two-sided, Student’s t-test. (H) Quantification of transepidermal water loss measurements between control and FibDTA mice at P10. n = 3 control and 3 FibDTA mice. p = 0.0790, ns, unpaired, two-sided, Student’s t-test.

Fibroblasts have a major role in producing and remodeling the extracellular matrix, including the basement membrane^16,17^. We assessed how collagen I, the major structural component of the dermal extracellular matrix, and the basement membrane, the substrate to which the epithelium is anchored, may be altered. Surprisingly, fibroblast depletion in neonatal mice does not lead to a statistically significant reduction in collagen I density as measured by second harmonic generation (SHG) mean fluorescence intensity (Figure 3A and C). These results suggest robust compensatory mechanisms maintain dermal structural integrity. Next, we interrogated basement membrane morphology and mechanics in fibroblast depleted neonatal mice. Morphologically, we find that the basement membrane is still intact by transmission electron microscopy when comparing control to fibroblast depleted mice (Figure 3B). To interrogate whether basement membrane mechanics may be perturbed despite appearing morphologically intact, we turned to atomic force microscopy (AFM) to measure basement membrane stiffness in frozen tissue sections (Figure 3D) and found significantly reduced basement membrane stiffness in fibroblast depleted mice compared to controls (Figure 3E).

As the stem cell layer is anchored to the basement membrane, we next asked whether fibroblast depletion could impact cell delamination from the epidermal stem cell layer. To examine this, we performed a 48-hour Edu chase experiment to track Edu^+^ epidermal differentiating stem cells that have reached the overlaying differentiated layer, the spinous layer, in control versus fibroblast depleted mice. A ratio of Edu^+^ cells in the spinous versus basal cell layer was used to estimate delamination from the basal layer. We find decreased delamination of epidermal differentiating stem cells in fibroblast depleted mice compared to controls (Figure 3F and G). These changes in basement membrane stiffness and epidermal differentiating stem cell delamination prompted us to assess overall barrier function by measuring transepidermal water loss (TEWL). We found no significant difference in TEWL measurements between fibroblast depleted mice compared to controls, indicating proper epidermal barrier function is maintained (Figure 3H).

Taken together, despite perturbations to basement membrane stiffness and epidermal stem cell delamination, neonatal dermal collagen density and epithelial barrier function are retained in the face of significant fibroblast depletion.

## Discussion

Fibroblasts are major contributors of organ integrity including through both secretion of critical structural elements and their ability to influence regeneration of neighboring epidermal stem cells^16,18,19^. Our results demonstrate that epidermal stem cell proliferation persists despite significant fibroblast depletion during both neonatal and adult stages, suggesting that the program to support epidermal regeneration is built in excess. These results parallel hair follicle studies which demonstrate that a critical number of dermal papilla fibroblasts are necessary to promote hair follicle epithelial stem cell growth^11^. This is not the case under conditions where the entire population of dermal papilla fibroblasts have been eliminated, which inhibits hair follicle growth entirely^10^. Moreover, reorganization of dermal papilla architecture leads to shorter hair production but still supports hair follicle stem cell proliferation and differentiation^20^. These results demonstrate that maintained presence of fibroblasts can still support stem cell growth even if architecture is altered.

In our system, a fraction of dermal fibroblasts remains after depletion, and epidermal stem cell proliferation continues, unmasking the potential for fibroblast compensation and redundancy in the dermis. This compensation is evident as neonatal fibroblast depletion does not lead to a significant decrease in fibroblast membrane coverage, demonstrating either that remaining fibroblasts hold the capacity to expand their membrane area or that the tissue is already built with intertwined architecture that can remain after depletion. Indeed, in aged mice, fibroblasts are lost in clusters, and also demonstrate membrane coverage compensation^12^. Maintenance of fibroblast membrane coverage may also aid the observed maintained collagen density between neonatal control and fibroblast depleted conditions. It is conceivable maintained membrane coverage ensures secretion is equally spread and that remaining fibroblasts augment their collagen I synthetic capacity to provide similar output generated by a larger fibroblast population.

Furthermore, results in this study reflect remarkable tissue capacity for compensation for critical physiological processes of other organ systems. For instance, intestinal brush border microvilli, which are essential for nutrient absorption, are supported by actin filaments and stabilized by actin bundling proteins. Remarkably, microvilli can still form despite knockout of three of the four known actin bundling proteins^21,22^, revealing a striking example of molecular redundancy to maintain tissue integrity.

Our study also complements observations of epidermal stem cell growth and differentiation in cell culture. These cell culture studies demonstrate a greater dependence of epidermal stem cells on fibroblasts for differentiation, whereas proliferation of epidermal stem cells is enhanced by the presence of fibroblasts but can continue over short periods or at high epidermal seeding densities in the absence of fibroblasts^4–7^. Our study examines epidermal proliferative capacity in acute stages (~1 week) following fibroblast depletion, and future studies should aim to understand whether the full repertoire of fibroblasts is necessary to sustain epidermal stem cell proliferation after longer periods following fibroblast depletion or under wounding conditions.

Overall, this work has important implications for the understanding of the regulation of epidermal stem cell behaviors in relation to dermal fibroblasts in live mice. Our work suggests that niche dependence may be consistent in skin appendages versus epidermis, underscoring the need for further comprehensive understanding of the complex interplay between different cell types in a physiological microenvironment.

## Methods

### Mice

The following mice were procured from the Jackson Laboratory: LSL-DTA (#009669)^23^, PDGFRα-CreER (#018280)^24^, mTmG (#037456)^14^ and PDGFRα-H2BGFP (#007669)^25^. To induce the depletion of fibroblast density, PDGFRα-CreER; LSL-DTA mice were compared to controls as indicated in figure legends, including Col1a1^flox/flox^ mice without PDGFRa-CreER, wherein loxp sites flank exons 2–5 of the COL1A1 (Mayumi Ito, New York University). To induce Cre recombination in neonatal mice, 50 μL of 3 mg/mL tamoxifen (80 μg/g) was administered intragastrically at P2. Mice from experimental and control groups were selected at random – independent of sex – at P10 and used for live imaging and tissue collection. To induce Cre recombination in adult mice, three dosages of 100 μg/g tamoxifen were given to 2-month-old mice intraperitoneally. One week later, mice from experimental and control groups were littermates selected at random – independent of sex – for tissue collection. No blinding was done. All procedures involving animal subjects were performed under the approval of the Institutional Animal Care and Use Committee (IACUC) of the Yale School of Medicine.

### *In vivo* imaging

Live imaging of mice was performed on the non-hairy, flat region of the right hind paws. Mice were anaesthetized by inhaling 2% isoflurane in oxygen and air. The hind paw was mounted on a custom-made stage with a warming pad and the mice remained anaesthetized with 1.5% isoflurane during imaging. A glass coverslip was placed against the region of interest with a water interface to minimize the vibration of breathing. Images were acquired with a LaVision Biotec (Miltenyi Biotec) two-photon microscope equipped with two tunable Ti:Sapphire lasers, a Chamelon Vision II (Coherent) (940 nm) and a Chameleon Discovery (Coherent) (1120 nm) through a 40X water immersion lens (NA 1.1; Zeiss or NA 1.15; Nikon) in 1 μm Z-steps. Mature collagen fibers capable of second harmonic generation (SHG) were excited by the 940 nm laser to emit signal collected in the blue channel.

### Immunofluorescence

For whole-mount staining, paw skin tissue was isolated and fixed in a 4% paraformaldehyde (PFA) solution in PBS overnight. The tissue was then transferred to an Eppendorf tube for blocking with 0.2% Triton X-100, 5% normal donkey serum, and 1% BSA in PBS. The tissue was incubated with primary antibodies for over 60 hours at room temperature, washed, and then incubated with secondary antibodies for over 60 hours at room temperature. The primary antibody used in this study were rabbit anti-pH3 (1:300; Millipore, 06–750). The secondary antibody used in this study were anti rabbit AlexaFluor 647 (1:200; Thermofisher). Where indicated, Hoechst 33342 (1:2000; Becton Cickinson: H3570), was added during the secondary incubation. Lastly, the tissues were mounted with Vectashield Anti-fade mounting medium (Vector Laboratories) or SlowFade Diamond Antifade Mountant (ThermoFisher) with an overlying coverslip sealed with nail polish. The slides were imaged on the LaVision two-photon microscope as described in “*In vivo* imaging” with 1–2 μm Z-steps. Whole mount image stacks were processed using FIJI to identify distinct layers of cells based on their proximity to the basal layer and the SHG signal in the dermis. Basal epidermal stem cells were identified based on their small and compact nuclei, while other layers were distinguished by their relative positions to the basal layer. The number of PH3-positive cells was quantified using FIJI’s cell counter. PH3^+^ cells were defined by large morphological “Bright Spots” overlapping with Hoechst signal.

### Electron Microscopy

The paw skin was dissected in freshly prepared 0.1 M sodium cacodylate buffer containing 4% PFA. The samples were transferred into fixation solution with 2% PFA and 2% glutaraldehyde in 0.1 M sodium cacodylate buffer at 37 °C for 30 min, room temperate for 2 hours, and 4 °C overnight. The fixed samples were moved to a storage buffer with 1 % PFA in 0.1 M sodium cacodylate buffer the next day. Prior to EM, samples were rinsed with 0.1 M sodium cacodylate buffer (pH 6.2), stained with 1% uranyl acetate and incubated in 70% ethanol. After dehydration through an ethanol series, the samples were embedded in araldite resin. Ultrathin (30–70 nm) sections were cut with a diamond knife using an ultramicrotome and placed onto copper grids for transmission electron microscopy (TEM). TEM was performed using a Zeiss 902A electron microscope (Zeiss). Images were acquired using the Megaview III Soft Imaging System camera and analySIS software (Soft Imaging System).

### Atomic Force Microscopy

For Atomic Force Microscopy (AFM), paw skin was dissected, nitrogen frozen and then embedded in OCT blocks. AFM measurements of the basement membrane were performed on freshly cut 20 μm cryosections using JPK Nano Wizard 2 (Bruker Nano) atomic force microscope mounted on an Olympus IX73 inverted fluorescent microscopy (Olympus) and operated via JPK SPM Control Software v.5. Cryosections were equilibrated in PBS supplemented with protease inhibitors and measurements were performed within 20 minutes of thawing the samples. Triangular non-conductive Silicon Nitride cantilevers (MLCT, Bruker Daltonics) with a nominal spring constant of 0,07 Nm^−1^ were used for the nanoindentation experiments of the apical surface of cells and nuclei. For all indentation experiments, forces of up to 3 nN were applied, and the velocities of cantilever approach and retraction were kept constant at 10 μm^s−1^ ensuring an indentation depth of 500 nm. All analysis were performed with JPK Data Processing Software (Bruker Nano). Prior to fitting the Hertz model corrected by the tip geometry to obtain Young’s Modulus (Poisson’s ratio of 0.5), the offset was removed from the baseline, contact point was identified, and the cantilever bending was subtracted from all force curves.

### EdU Treatment and Quantification

EdU, 5-ethynyl-2’-deoxyuridine, is a thymidine analog that incorporates into DNA. An EdU pulse chase experiment was adopted to examine the differentiation process of the epidermis and epidermal proliferative capacity^26^. To label basal cells, neonatal mice were pulsed with EdU (50 μg/g in PBS) via intraperitoneal injection. For the 48 hour Edu chase, mice were pulsed at P8, and 48 hours after the injection at P10, paw skin tissues were isolated and fixed in 4% aqueous PFA solution overnight. For the 6 hour Edu chase, mice were pulsed at P10 and paw skin tissues were isolated and fixed 6 hours after injection in 4% aqueous PFA solution overnight. EdU incorporation was evaluated by the Click-iT EdU Cell Proliferation Kit for Imaging Alexa Fluor 488 dye (48 hour Edu Chase, Invitrogen C10337) or Alexa Fluor 647 dye (6 hour Edu chase, C10340) based on the manufacturer’s instructions. The tissues were then stained as described in “whole-mount staining”. After image acquisition, the delamination status of EdU positive cells were expressed as the distance from basal cells manually analyzed in FIJI using the line measurement for the 48 hour Edu chase. For the 6 hour Edu chase, Edu^+^ cells were counted and plotted as a percentage of total basal epidermal stem cell nuclei.

### Cytokine assay

Lipopolysaccharide (LPS) was injected intraperitoneally at 2 μg/g in CD1 mice at P9 as a positive control for inflammation. Blood samples from P9 mice of respective treatments including controls were collected from the neck at the time of decapitation. The samples were spun-down, and serum was collected and kept at −80°C until the assay was performed. To assess inflammatory cytokines and growth factor cytokines in our models, we performed a Bio-Plex ProTM Mouse Cytokine 8-Plex Assay and Bio-Plex ProTM Mouse Cytokine 9-Plex Assay respectively (Bio-rad laboratories; M60000007A, MD000000EL). The assays were performed according to the manufacturer’s instructions. Briefly, serum samples were thawed and diluted 1:4 with provided diluent. 50 μL of each sample including standards and blanks were added to the 10X coupled bead pre-coated plate and incubated for 30 mins on a shaker at room temperature. The samples were washed three times and incubated with detection antibody for an additional 30 minutes on a shaker at room temperature, washed three times and incubated with SA-PE for 10 minutes. The samples were then washed for a final three times and resuspended in 125 μL of assay buffer before running on a Luminex 200 analyzer with a dual laser detection platform.

### Fluorescence Activated Cell Sorting

P10 and adult paws were collected and processed into single cell suspensions using an adapted protocol from Yang et al. (2017, Cell 169, 483–496). Specifically, paws were placed dermis side down in 0.25% collagenase IV (Sigma; C5138) in HBSS (GIBCO;14170–112) for 45 minutes followed by 20 minutes in 0.25% trypsin (GIBCO; X). After digestion, the paws were scraped using a blunt blade to create single cell suspensions and washed in FACS Buffer (3% FBS, 2mM EDTA). The suspensions were then centrifuged at 350G for 10 minutes and filtered through a 40μm filter (Falcon; 352340) before staining. The following antibodies were used for staining. Fibroblast heterogeneity: CD26-PE (Biolegend;137803), Sca-1 APC (Invitrogen;17–5981-83). Immune profiling: CD45-FITC (Biolegend;147710), Ly-6G-PE-efluor^™^610 (Invitrogen; 61–9668-82), CD11b-APC (Invitrogen; 17–0112-82), CD45-PE (Biolegend;137803), F4–80- APC (Biolegend; 123116), CD3 APC (Biolegend; 100236). Cells were acquired on a Becton Dickinson LSRII Flow Cytometer outfitted with Diva software v 8.0.1, and the data was analyzed using Flowjo v.10.9.0.

### Imaging Analysis

Raw live imaging data was imported into FIJI (ImageJ, NIH) for stitching^27^. To reduce the dimensionality of 3D imaging stacks we used Imaris software (Oxford Instruments) to isolate the upper dermal signal and a custom MATLAB script (MathWorks) to flatten it. Upon importing the image stack into Imaris, the dermal space was defined by creating a surface from the SHG collagen signal. Using Imaris’ distance transformation tool, a new channel was created to represent distance from the dermal surface. A third surface, an inverse of the original dermal surface, was used to generate a second distance transformation channel representing distance into the dermis. This allowed us to mask a defined distance into the dermis from the epidermal-dermal interface. The resulting masked channels were exported back to FIJI and run through our custom MATLAB script for flattening and generation of maximum intensity projections for further MFI analysis. Consistent thresholding in Fiji was applied to all maximum intensity projections to quantify the relative % membrane coverage based on area.

The adult fibroblast nuclei count was conducted using Imaris software. The PDGFRaH2BGFP signal was imported into Imaris as a z-stack. Next, the Imaris spot function was used to identify nuclei spots, with a spot diameter of 7 μm. Any inaccuracies in thresholding were manually corrected in Imaris before exporting statistical data for the final nuclei count.

### Trans-epidermal water loss measurement

Barrier function was determined using a Tewameter TM Nano (Courage and Khazaka electronic). P10 mice were anaesthetized by inhaling 2% isoflurane in oxygen and air and then probed on the paw to measure trans-epidermal water loss. The data was acquired using the CK-MPA software provided by the manufacturer. The data was then exported to excel for analysis.

### Statistics and reproducibility

Biostatistical analyses were performed with the GraphPad Prism (Version 9.4) software (GraphPad Inc., La Jolla, CA). The distribution of replicates was described by means with standard deviations in the graphs where indicated. Statistical comparisons between conditions were made using the unpaired two-tailed Student’s t-test or Kolmogorov-Smirnov test where indicated.

### Data availability

All the data that support the findings of this study are available from the corresponding author upon reasonable request.

## Supplementary Material

Supplement 1
