# Supplementary material for: Fibroblast depletion reveals mammalian epithelial resilience across neonatal and adult stages
Source: bioRxiv. 2025 Jul 31:2025.07.29.667423. Preprint. [Version 1] doi: 10.1101/2025.07.29.667423 (PMC12324404; doi:10.1101/2025.07.29.667423)
Supplement: Supplementary file 1 [file media-1.pdf]

Supplementary Figure 1

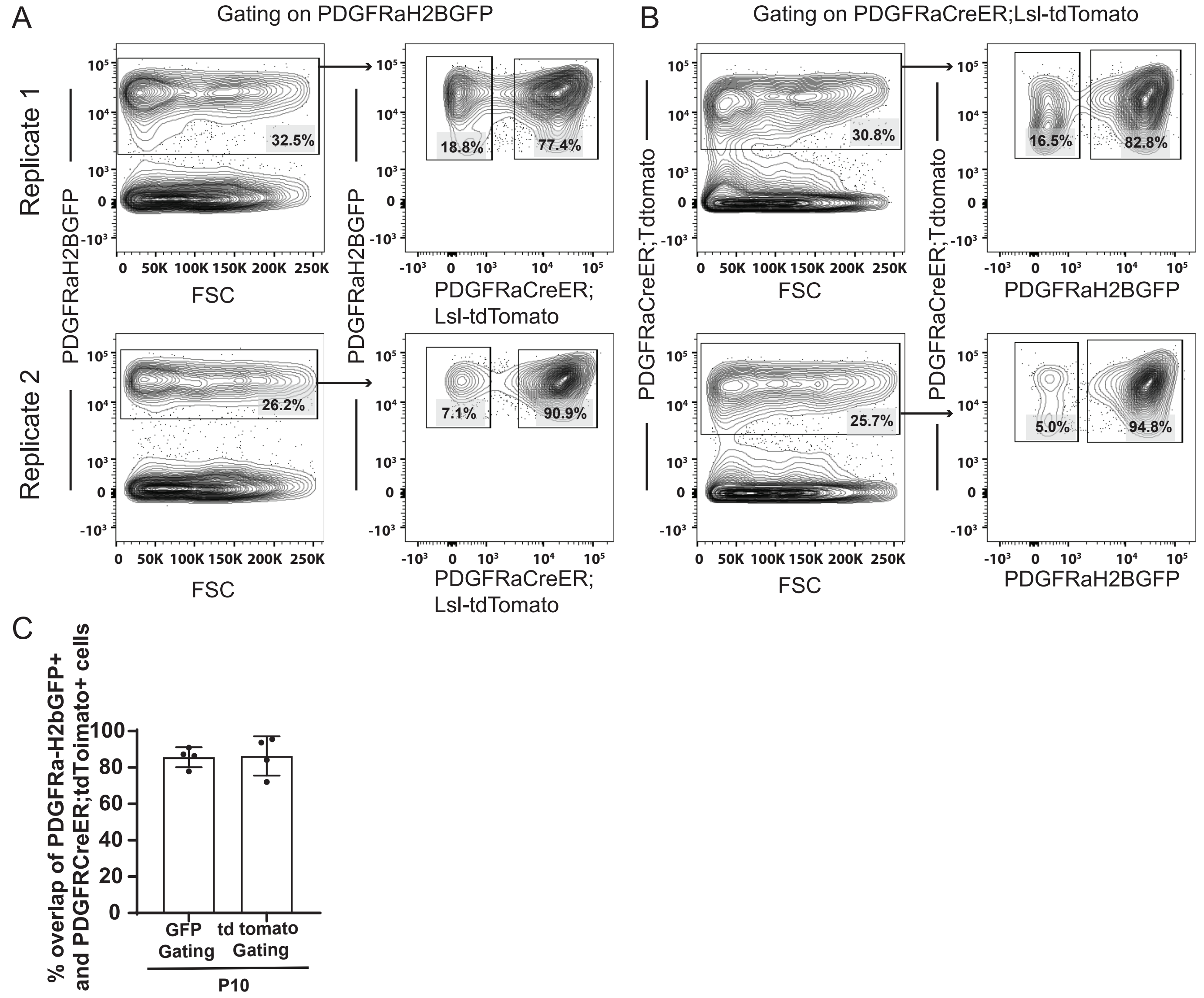

Supplementary Figure 1.

(A) Representative FACS plots from two replicate P10 mice expressing PDGFRα-H2B-GFP; PDGFRα-CreER; lsl-tdTomato gating first on GFP+ cells and then tdTomato+ to determine the extent of the overlap (GFP+tdTomato+ dermal cells) between the two models. n = 4 mice.

(B) Representative FACS plots from two replicates from (A) expressing PDGFRα-H2B-GFP; PDGFRα-CreER; lsl-tdTomato gating first on tdTomato+ cells and then GFP+ to determine the extent of the overlap (GFP+tdTomato+ dermal cells) between the two models. n = 4 mice.

(C) Quantification of the percent overlap of cells expressing both PDGFRα-H2B-GFP and PDGFRα-CreER; LSL-tdTomato, showing an average of 86% overlap regardless of the gating strategy. n= 4 mice p= 0.9085, ns, unpaired, two-sided Student's t-test.

Supplementary Figure 2

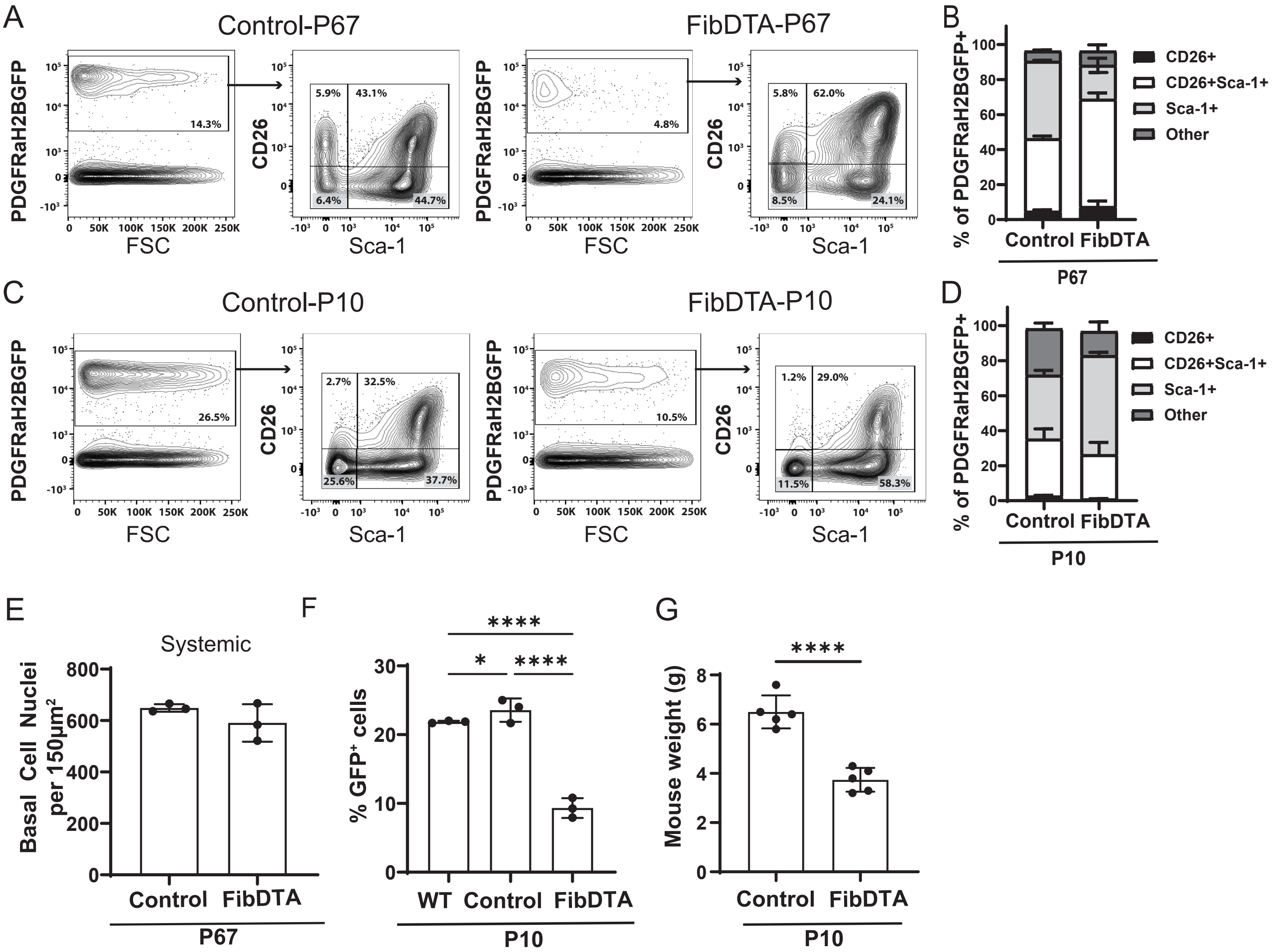

Supplementary Figure 2.

(A) Representative FACS plots showing fibroblast heterogeneity in P67 mice. n=3 control (PDGFRα-H2B-GFP; LSL-DTA) mice compared to n= 3 FibDTA (PDGFRα-H2B-GFP; PDGFRα-CreER ;LSL-DTA) mice after systemic depletion of PDGFRα+ cells. Cells were gated on GFP first, and then gated on CD26+Sca-1-, CD26+Sca-1+, CD26-Sca-1+ and CD26-Sca-1- (Other).

(B) Quantification of fibroblast heterogeneity in P67 mice. n=3 control (PDGFRα-H2B-GFP; LSL-DTA) mice compared to n=3 FibDTA (PDGFRα-H2B-GFP; PDGFRα-CreER ;LSL-DTA) mice after systemic depletion of PDGFRα+ cells. **p = 0.0016 (CD26+), p = 0.2054 ns (CD26+Sca-1+), ***p=0.0007 (CD26-Sca-1+), *p=0.0496 (CD26-Sca-1-, Other), unpaired, two-sided, Student's t-test.

(C) Representative FACS plots showing fibroblast heterogeneity in P10 mice. n=3 control (PDGFRα-H2B-GFP; LSL-DTA) mice compared to n=3 FibDTA (PDGFRα-H2B-GFP; PDGFRα-CreER ;LSL-DTA) mice after systemic depletion of PDGFRα+ cells. Cells were gated on GFP first, and then gated on CD26+Sca-1-, CD26+Sca-1+, CD26-Sca-1+ and CD26-Sca-1- (Other).

(D) Quantification of fibroblast heterogeneity in P10 mice. n=3 control (PDGFRα-H2B-GFP; LSL-DTA) mice compared to n=3 FibDTA (PDGFRα-H2B-GFP; PDGFRα-CreER ;LSL-DTA) mice after systemic depletion of PDGFRα+ cells. p = 0.2705 ns (CD26+), *p = 0.0164 (CD26+Sca-1+), **p=0.0013 (CD26-Sca-1+), p=0.3526 ns (CD26-Sca-1-, Other), unpaired, two-sided, Student's t-test.

(E) Quantification of epidermal stem cell density in control and FibDTA adult mice after systemic tamoxifen induction. Graph represents average number of Hoecsht-positive cells within a 150 μm^2 area with SD from n = 3 mice. $^{ns}p = 0.2462$, unpaired, two-sided, Student's t-test.

(F) Quantification of fibroblast depletion in P10 neonatal mice by FACS, comparing CD1 WT (WT), PDGFRα-H2B-GFP;lsl-DTA (Control) and PDGFRα-H2B-GFP; PDGFRα-CreER; lsl-DTA FibDTA mice. n = 3 mice per group. ****p<0.0001 unpaired, two-sided, Student's t-test when comparing WT to FibDTA and Control to FibDTA. *p<0.0186 unpaired, two-sided, Student's t-test when comparing WT to Control.

(G) Quantification of mouse weights in n = 5 control (LSL-DTA) and 5 FibDTA (PDGFRα-CreER;LSL-DTA) mice. ****p<0.0001 unpaired, two-sided, Student's t-test.

Supplementary Figure 3

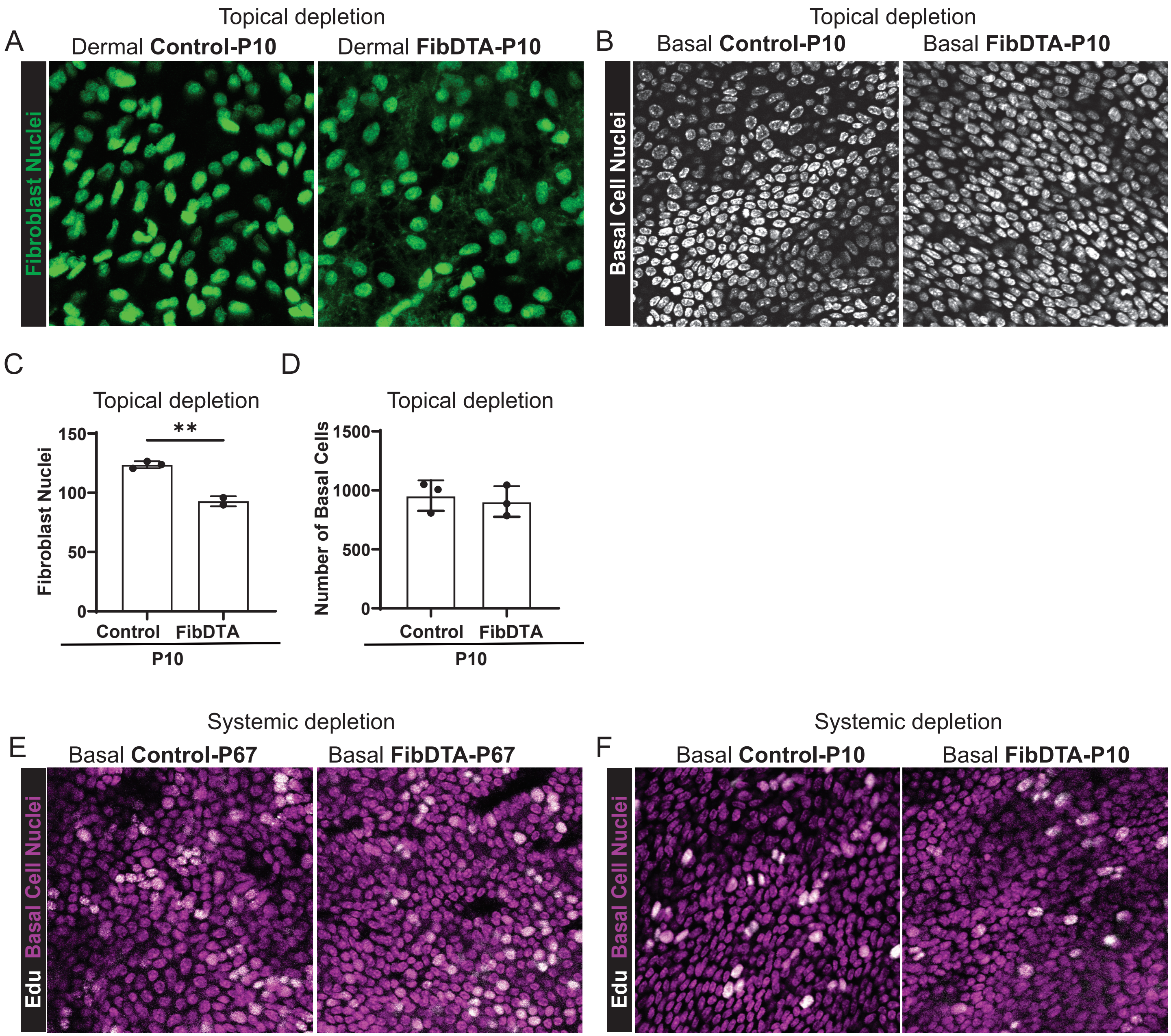

Supplementary Figure 3.

(A) Representative images of fibroblast depletion in P10 paw skin following topical administration of 4-OHT in control (lsl-DTA;mTmG) or FibDTA (lsl-DTA; PDGFRα-CreER; mTmG).

(B) Representative images of epidermal stem cell nuclei in control and FibDTA mice following topical administration of 4-OHT.

(C) Quantification of fibroblast number identified by the PDGFRα-H2BGFP signal in control and FibDTA in P10 mice following topical administration of 4-OHT. n = 3 control and n = 2 FibDTA mice. **p = 0.0022, unpaired, two-sided, Student's t-test.

(D) Quantification of epidermal stem cell number in control and FibDTA P10 mice following topical administration of 4-OHT. n= 3 control and 3 FibDTA mice. p = 0.6649, ns, unpaired, two-sided, Student's t-test.

(E) Representative whole mount images of control and FibDTA mice at P67 after 6hr Edu chase. Epidermal stem cells are represented in magenta and Edu+ cells in white.

(F) Representative whole mount images of control and FibDTA mice at P10 after 6hr Edu chase. Epidermal stem cells are represented in magenta and Edu+ cells in white.

Supplementary Figure 4

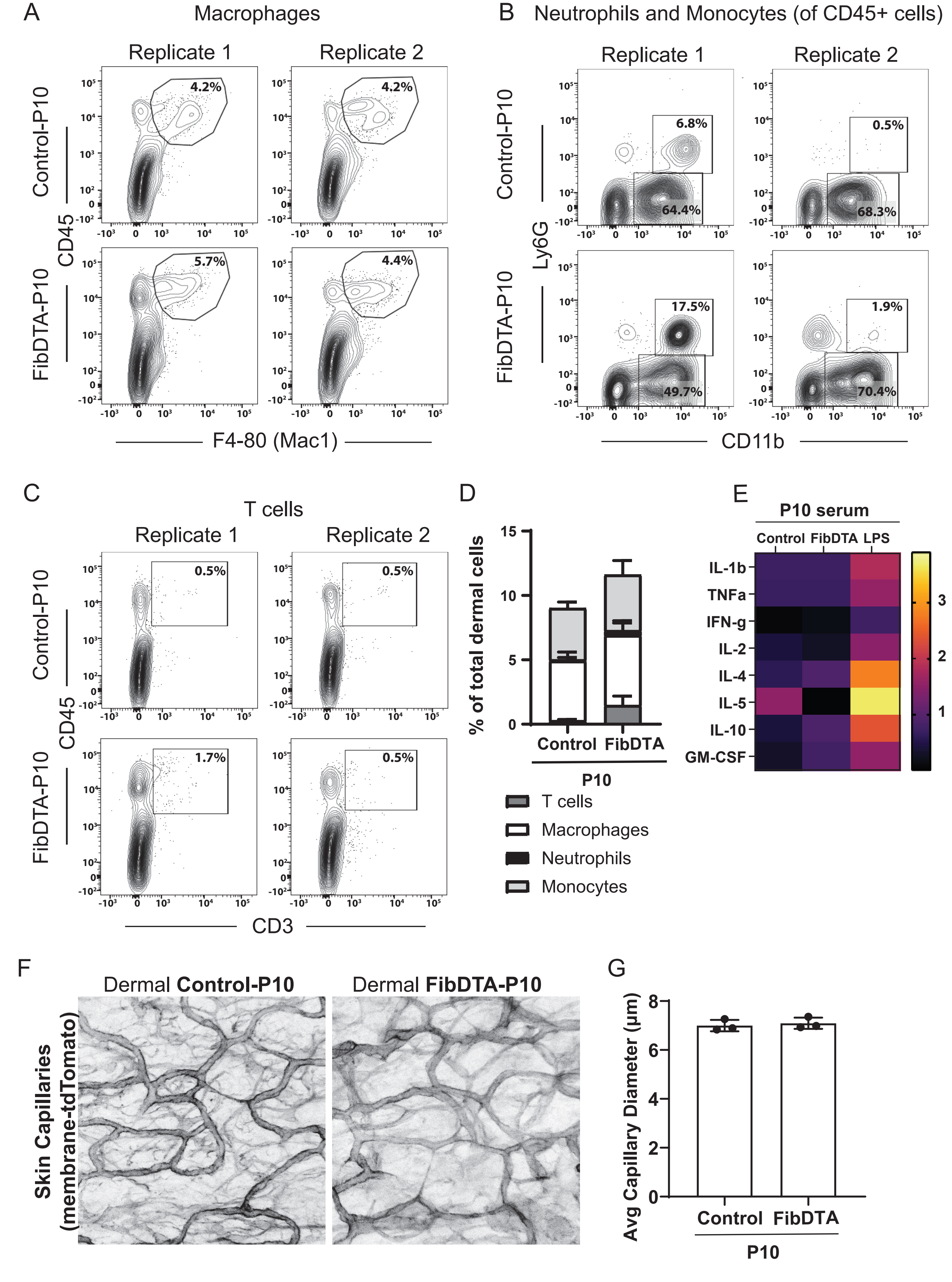

Supplementary Figure 4.

(A) Representative flow cytometry plots showing macrophage percentages in dermal preparations of P10 mice from a total of n=3 control (PDGFRα-H2B-GFP; LSL-DTA) mouse paw samples compared to n=4 FibDTA (PDGFRα-H2B-GFP; PDGFRα-CreER ;LSL-DTA) after systemic depletion of PDGFRα+ cells. Cells were gated on CD45 and F4-80.

(B) Representative flow cytometry plots showing neutrophil (Ly6G+Cd11b+) and monocyte (CD11b+) percentages in dermal preparations of P10 mice from a total of n = 3 control (PDGFRα-H2B-GFP; LSL-DTA) mouse paw compared to n=4 FibDTA (PDGFRα-H2B-GFP; PDGFRα-CreER ;LSL-DTA) mice after systemic depletion of PDGFRα+ cells. Cells were gated first on CD45 and then on LY6G and CD11B.

(C) Representative flow cytometry plots showing T cell percentages in dermal preparations of P10 mice from a total of n = 3 control (PDGFRα-H2B-GFP; LSL-DTA) mouse paw compared to n=4 FibDTA (PDGFRα-H2B-GFP; PDGFRα-CreER ;LSL-DTA) after systemic depletion of PDGFRα+ cells. Cells were gated on CD45 and CD3.

(D) Quantification of different immune populations in dermal preparations of P10 mice. n= 3 control (PDGFRα-H2B-GFP; LSL-DTA) mouse paw compared to n=4 FibDTA (PDGFRα-H2B-GFP; PDGFRα-CreER ;LSL-DTA) after systemic depletion of PDGFRα+ cells shown in (A-C). p = 0.2689, ns (macrophages), p = 0.6610, ns (monocytes), p = 0.5584 ns (neutrophils),*p = 0.0.039, unpaired, two-sided, Student's t-test.

(E) Normalized heatmap of cytokine panel taken from serum of P10 mice. n = 3 control, n = 3 FibDTA and n = 2 LPS treated mice.

(F) Representative images of the skin capillary plexus, determined by morphology and labeled with mTmG in P10 control and FibDTA mice.

(G) Average capillary diameter measurement for n = 3 control (PDGFRα-CreER; mTmG) and and n = 3 FibDTA (PDGFRα-CreER; mTmG; LSL-DTA) P10 mice, $p = 0.6435$, ns, unpaired, two-sided, Student's t test.
